# Increasing value in research: cost evaluations in health professions education

**DOI:** 10.1111/medu.14007

**Published:** 2019-11-21

**Authors:** Sanne Schreurs, Kitty Cleutjens, Mirjam G A oude Egbrink

**Affiliations:** ^1^ Department of Educational Development and Research School of Health Professions Education Faculty of Health, Medicine and Life Sciences Maastricht University Maastricht the Netherlands; ^2^ Department of Pathology Faculty of Health, Medicine and Life Sciences Maastricht University Maastricht the Netherlands; ^3^ Department of Physiology Institute for Education and School of Health Professions Education Faculty of Health, Medicine and Life Sciences Maastricht University Maastricht the Netherlands

## Abstract

The authors use Foo et al.'s discussion of the value of economic evaluations to consider how such techniques might advance the practice of selection for medical school.

Nowadays, higher education institutes have to deal with a combination of limited financial resources and increasing demands for accountability. As a result, considerations of costs and value have become paramount in stakeholders’ choices. This also holds for health professions education (HPE): imagine a department proposing to buy a high‐quality spinning bike because they find it important and interesting to use this in practical sessions on energy expenditure during exercise for medical students. Ideally, the medical school's management should make a well‐informed decision regarding spending the required amount of money to buy this device or saving it for other educational activities. To enable such a decision, they should have information on whether this financial investment is balanced by increased student learning. However, this information is often not available: studies on costs are extremely scarce in HPE and studies including value are even more rare. Furthermore, the systematic review by Foo and colleagues in this issue of Medical Education^1^ concluded that the quality of most of the available research is substandard and has not increased since the beginning of this century. They found that shortcomings in the cost‐evaluation literature are mostly related to the methodology and reporting specific to these analyses. This is, at least in part, caused by a lack of expertise in performing economic evaluations within the field of HPE. In addition, Foo and colleagues show that very few studies reporting cost data in HPE represent full economic evaluations of educational activities, in which costs and value of two or more approaches are evaluated and contrasted.^1^ These findings mirror the practice of higher education institutes: decisions on which educational activities to invest in are often not fuelled by full empirical economic evaluations.

These findings mirror the practice of higher education institutes: decisions on which educational activities to invest in are often not fuelled by full empirical economic evaluations

One educational area in which cost evaluations are particularly interesting is that of selection for medical school. Over the past few decades, many different selection tools have been developed, employed and combined into different selection procedures at various medical schools.^2^ It is as if every school reinvented the wheel, because ‘track and weather’ conditions are slightly different everywhere. Medical schools are under increasing pressure to justify their unique and often expensive selection procedures in terms of costs and value. However, research on these costs and especially the value of selection procedures, or even the tools within these procedures, is sorely lacking.^3^ Therefore, not only is each medical school using a different set of wheels, but importantly, the price tag on these wheels is blank. Furthermore, because research in the field of selection has focused almost solely on the predictive and incremental value of different tools, there is barely any information on the construct validity of selection procedures, as defined in the modern validity theories.^4^, ^5^ Research has focused on supporting the ‘relation to other variables’ (e.g. predictive validity) of selection tools, severely neglecting the other sources of evidence for validity (i.e. content, response processes, internal structure and consequences^6^, ^7^). Thus, selection wagons are running on different wheels, and we do not know what they cost nor how crooked they are. This was already observed in 1998 by Tekian: ‘To begin to understand the nuts and bolts of the admission machine, we opened the gear box, but only to find a jumble of parts that prevents the machine from running smoothly’.^8^ In an attempt to get the wagons running smoothly, research in the field of selection for medical school should move towards a more general understanding of validity. Importantly, the ‘consequences’ pillar of validity should be taken into account explicitly, and must include costs as well as value related to selection tools and procedures, preferably also in comparison with other tools or procedures, as in cost–benefit evaluations.^1^

Medical schools are under increasing pressure to justify their unique and often expensive selection procedures in terms of costs and value

… selection wagons are running on different wheels, and we do not know what they cost nor how crooked they are

Some rare examples of cost evaluations in the field of selection do exist, for example, on how many stations are needed in a multiple mini‐interview to achieve a certain reliability and acceptability.^9^ In an effort to start the cost‐and‐value discussion on selection procedures as a whole, we have recently investigated the costs of the selection procedure at Maastricht University Medical School, and contrasted this with an inexpensive, weighted lottery procedure. We also looked into the value these procedures returned and concluded that although our tailor‐made selection procedure was much more expensive to conduct than the lottery procedure, it already paid itself back in terms of value during the three pre‐clinical years of medical school alone.^10^ In our study, we used the CHEERS statement^11^ to safeguard the quality of the study. Nevertheless, in line with Foo et al.'s conclusions,^1^ we experienced challenges such as using the right nomenclature, choosing the correct methodology and putting a price on the value‐side of selection. The recently published Association for Medical Education in Europe guide on how to read studies on educational costs^12^ and some exemplary studies (e.g. Foo et al.^13^) are highly welcomed aids to support the process of setting up, conducting and reporting future economic evaluations in HPE. Moreover, Foo et al.'s suggestion to involve experts in health or educational economics at the earliest possible stage is essential to improve the quality of cost‐evaluation research.^1^

… Foo et al.'s suggestion to involve experts in health or educational economics at the earliest possible stage is essential to improve the quality of cost‐evaluation research

Importantly, high‐quality cost‐evaluation research is not only relevant at the level of individual educational institutes, but also on a national level. As an example, we refer to the situation in the Netherlands: a few years ago, medical schools were obliged to switch from a national weighted lottery to decentralised selection procedures, which differ from school to school. Because several of these procedures are insufficiently predictive to warrant the monetary investments (see e.g. in Wouters^14^ ) the cost‐effectiveness of selection in general is questioned and reintroduction of a national lottery system is asked for. This dispute has even sparked discussion at the government level. In our opinion, research into the general construct validity of the selection procedures, including high‐quality full economic evaluations, is crucial to support an evidence‐based governmental decision on this issue.

Importantly, high‐quality cost‐evaluation research is not only relevant at the level of individual educational institutes, but also on a national level

All in all, there is a lack of high‐quality economic evaluations in HPE in general and selection in particular. This puts the stakeholders in a precarious position, in which they have to remain accountable regarding their limited financial resources when choosing between educational activities without price tags. Therefore, educational researchers should join efforts to conduct more economic evaluations to provide relevant stakeholders with economically valid arguments to make well‐informed decisions that benefit the quality of education.
